# Transforming growth factor receptor III (Betaglycan) regulates the generation of pathogenic Th17 cells in EAE

**DOI:** 10.3389/fimmu.2023.1088039

**Published:** 2023-02-06

**Authors:** Samuel J. Duesman, Sandra Ortega-Francisco, Roxana Olguin-Alor, Naray A. Acevedo-Dominguez, Christine M. Sestero, Rajeshwari Chellappan, Patrizia De Sarno, Nabiha Yusuf, Adrian Salgado-Lopez, Marisol Segundo-Liberato, Selina Montes de Oca-Lagunas, Chander Raman, Gloria Soldevila

**Affiliations:** ^1^ Department of Dermatology, University of Alabama at Birmingham, Birmingham, AL, United States; ^2^ Department of Immunology, Biomedical Research Institute, National Autonomous University of Mexico (UNAM), Mexico City, Mexico; ^3^ National Laboratory of Flow Cytometry, Biomedical Research Institute, National Autonomous University of Mexico (UNAM), Mexico City, Mexico; ^4^ Department of Biology, Chemistry, Mathematics and Computer Science, University of Montevallo, Montevello, AL, United States; ^5^ Department of Pathology, University of Alabama at Birmingham, Birmingham, AL, United States; ^6^ Department of Biology, University of Alabama at Birmingham, Birmingham, AL, United States

**Keywords:** autoimmunity, EAE, pathogenic Th17, Th1, IFN-g, polarization

## Abstract

The transforming growth factor receptor III (TβRIII) is commonly recognized as a co-receptor that promotes the binding of TGFβ family ligands to type I and type II receptors. Within the immune system, TβRIII regulates T cell development in the thymus and is differentially expressed through activation; however, its function in mature T cells is unclear. To begin addressing this question, we developed a conditional knock-out mouse with restricted TβRIII deletion in mature T cells, necessary because genomic deletion of TβRIII results in perinatal mortality. We determined that TβRIII null mice developed more severe autoimmune central nervous neuroinflammatory disease after immunization with myelin oligodendrocyte peptide (MOG_35-55_) than wild-type littermates. The increase in disease severity in TβRIII null mice was associated with expanded numbers of CNS infiltrating IFNγ^+^ CD4^+^ T cells and cells that co-express both IFNγ and IL-17 (IFNγ^+^/IL-17^+^), but not IL-17 alone expressing CD4 T cells compared to *Tgfbr3^fl/fl^* wild-type controls. This led us to speculate that TβRIII may be involved in regulating conversion of encephalitogenic Th17 to Th1. To directly address this, we generated encephalitogenic Th17 and Th1 cells from wild type and TβRIII null mice for passive transfer of EAE into naïve mice. Remarkably, Th17 encephalitogenic T cells from TβRIII null induced EAE of much greater severity and earlier in onset than those from wild-type mice. The severity of EAE induced by encephalitogenic wild-type and *Tgfbr3^fl/fl^.dLcKCre* Th1 cells were similar. Moreover, *in vitro* restimulation of *in vivo* primed *Tgfbr3^fl/fl^.dLcKCre* T cells, under Th17 but not Th1 polarizing conditions, resulted in a significant increase of IFNγ^+^ T cells. Altogether, our data indicate that TβRIII is a coreceptor that functions as a key checkpoint in controlling the pathogenicity of autoreactive T cells in neuroinflammation probably through regulating plasticity of Th17 T cells into pathogenic Th1 cells. Importantly, this is the first demonstration that TβRIII has an intrinsic role in T cells.

## Introduction

Transforming growth factor receptor III (TβRIII), also known as Betaglycan, is a surface proteoglycan that is broadly expressed in many tissues and cell types (1). It is involved in multiple cellular processes including activation, proliferation, adhesion, migration, and organogenesis (2–4); therefore, *Tgfbr3 knockout* mice die before E18 embryonic stage (5). Most recently, TβRIII has taken relevance in cancer progression, as its loss promotes epithelial-mesenchymal transition and enhances the migratory potential of malignant cells, being proposed as an important marker for metastasis prognosis (6–9). The TGFβ superfamily is composed of more than 30 ligands that can be classified into 3 subfamilies: activins and inhibins, bone morphogenic proteins (BMP’s), and TGFβs (including TGFβ1, 2, and 3). The canonical pathway triggered by these ligands involves their binding to TβRII and the subsequent recruitment of TβRI, leading to the phosphorylation of R-SMAD proteins and the activation of the common SMAD protein (SMAD4) which is then translocated to the nucleus to regulate gene expression.

TβRIII has been classically described as a co-receptor promoting the binding of all three isoforms of TGFβ to TβRII (1, 10, 11). TβRIII also binds inhibins, BMPs and Fibroblast Growth Factor (2, 11). In addition to promoting high affinity binding of TGFβ to TβRI and TβRII, TβRIII can also be cleaved from the cell membrane where it binds soluble TGFβ, thereby acting as a ligand trap (12). Considering the key role of TGFβ and other ligands of the TGFβ superfamily in T cell biology (13–15), it is not surprising that TβRIII has been described as an important regulator of T cell maturation and differentiation. This receptor is important for the survival and differentiation of T cells in the thymus, as fetal thymocytes lacking TβRIII exhibit higher apoptosis and a delay in T cell development (16); therefore it is reasonable to think that TβRIII would function as a regulator of mature T cell proliferation and differentiation. In a previous work, we demonstrated that TβRIII is expressed on T cells, highly expressed on CD4^+^ T cells (preferentially on naïve and central memory CD4^+^ T subpopulations) compared to CD8^+^ T cells, but not on B cells, both in mouse and human (17). Additionally, TβRIII was upregulated after TCR stimulation, with similar expression kinetics to other activation markers such as CD25, CD69, and CD44. Interestingly, both thymic and induced Tregs expressed very low levels of TβRIII, as Foxp3 expression is associated with a decreased expression of TβRIII (17). These data suggest that TβRIII may play an important function during T cell activation and/or during TGFβ mediated lineage differentiation.

The activation and differentiation of T cells is a highly regulated process involving several signaling pathways, and under certain circumstances the lack of signal regulation triggers pathologies, such as autoimmune diseases. In this context, TGFβ signaling is crucial as TβRI deficiency in CD4 cells in mice results in the development of spontaneous autoimmunity in mice (18). Similarly, when TβRII is deleted in CD4^+^ T cells, mice develop lethal inflammation and multi-organ autoimmune inflammatory infiltration (19), however the role of TβRIII in autoimmunity is not yet described.

Experimental autoimmune encephalomyelitis (EAE), though imperfect, is the best model to study immunopathogenic mechanisms of human multiple sclerosis (MS) (20, 21). CD4^+^ T cells reactive to myelin antigens initiate the disease and both Th1 and Th17 cells mediate an auto-inflammatory response which results in demyelination and axonal damage in the central nervous system leading to ascending paralysis (22). The developmental of EAE and severity of disease is sensitive to factors that tune thresholds of T cell activation, as well as those that have an impact on differentiation to effector populations. Simply stated, enhanced CD4^+^ T cell activation, such as attenuation of inhibitory signals or augmentation of co-stimulation will lead to increased disease severity. Conversely, factors that attenuate T cell activation such as increased inhibitory signals or loss of co-stimulation will decrease EAE severity. Factors that promote differentiation to Th1 cells and pathogenic Th17 cells also enhance EAE severity. IL-6 and TGF-β are key first-step cytokines involved in the differentiation of naïve CD4^+^ T cells to Th17 cells followed by IL-23 that promotes stability and pathogenicity of Th17 cells (23, 24). Additionally, at least *in vitro*, low concentrations of TGF-β promote generation of inflammatory Th17 while strong TGF-β signaling will shift balance to regulation (13, 25). After exposure to certain cytokine (IL-23 and IL-12) cues and signals, Th17 cells can also undergo a process described as plasticity that leads transdifferentiation to IFN-γ expressing Th1-like cells through an intermediate that expresses both IL-17 and IFN-γ (Th17/1 cells) (26, 27). The transdifferentiation promotes pathogenicity in EAE. During this transdifferentiation process, Th17 cells gain the expression of Tbet and may downmodulate expression of the master regulator RORγt (28). Severity of EAE and by extension progression of MS disease will be impacted by molecules that modulate Th17 pathogenicity and transdifferentiation.

To interrogate the function of TβRIII in T cells, we generated a mature T cell specific loss of function mutant mouse. Using this novel mouse, we show that T cell specific TβRIII null mice develop normally, have no obvious alterations in peripheral T cell populations and they respond equivalent to wild type to *in vitro* activation and CD4^+^ effector T cell differentiation. Induction of EAE in TβRIII null mice leads to greater severity of disease that is associated with expansion of IFN-γ and Th17/1 cells but not Th17 in the central nervous system (CNS). However, encephalitogenic TβRIII null Th17 cells but not Th1 cells are more pathogenic in the context of ability to induce EAE in naïve recipient mice. Overall, our data uncovers a biology of CD4^+^ T cell specific expression of TβRIII in restraining Th17 pathogenicity.

## Materials and methods

### Mice

*Tgfbr3^fl/fl^* mice were generated at UAB transgenic/ES core facility using ES cells obtained from EUCOMM. B6.Cg-Tg(Lck-icre)3779Nik/J (*dLckCre*) were obtained from JAX and bred to *Tgfbr3^fl/fl^* mice to generate *Tgfbr3^fl/fl^.dLckCre* and littermate *Tgfbr3^fl/fl^* mice. Female and male eight- to twelve-week-old littermate mice were used in all the experiments. All animals were bred and maintained in the animal facility at the University of Alabama and the Instituto de Investigaciones Biomédicas (IIB, UNAM, Mexico), in specific pathogen-free conditions, according to the ethical guidelines from the National Institute of Health, the Committee of Animal Care and Use at UAB and the Comité para el Cuidado y Uso de Animales de Laboratorio (CICUAL) at the IIB UNAM (protocol #176), that describe methods of sacrifice, methods of anesthesia and/or analgesia, and efforts to alleviate suffering.

### Assessment of *Tgfbr3* deletion

The deletion of *Tgfbr3* Exon 5 was assessed by PCR on DNA isolated from T CD4^+^ cells and compared with CD4-depleted splenocytes using the following primers: *forward (*TGF5FOR1) TGTTGTGGTGACTGTTGGCA and *reverse* (TGFEX1FOR1) GTTTCGGAGGGTTCTGTGGT. PCR was performed using the AmpliTaq Gold polymerase (Thermo fisher) following the manufacturer’s instructions.

To evaluate the loss of TβRIII expression on peripheral T cells, we used a goat anti-mouse TβRIII antibody followed by incubation with a donkey anti-goat IgG AF488 secondary antibody on live CD4^+^, CD8^+^, and CD19^+^ cells. Specifications of antibodies used can be found on **Supplementary Material (Table 1)** . Secondary antibody (Ab) staining was used as negative control for both thymocytes and splenocytes from *Tgfbr3^fl/fl^.dLckCre* and *Tgfbr3^fl/fl^* mice. Samples were acquired in the NxT cytometer (Thermo fisher) and analyzed with FlowJo X software (BD, San Jose, CA).

### Analysis of lymphocyte subpopulations

Spleen, mesenteric lymph nodes, peripheral lymph nodes, and blood samples were obtained from *Tgfbr3^fl/fl^.dLckCre* and *Tgfbr3^fl/fl^* mice. Cells were homogenized, counted, and 1x10^6^ cells were stained with different antibody panels to analyze B cells (CD19^+^), T cells (CD4^+^ and CD8^+^) and among these, naïve T cells (CD44^lo^ CD62L^hi^ CCR7^+^), central memory (CD44^hi^CD62L^+^CCR7^+^), and effector memory T cells (CD44^hi^CD62L^-^CCR7^-^). The complete list of antibodies is shown in **Supplementary Material (Table 1)**. Briefly, antibodies were diluted in PBS and cells stained with 50**μ**L of the antibody mix for 30 min at 37°C (for optimal staining of CCR7). Then, cells were washed with PBS and fixed with a 4% paraformaldehyde solution for 5 min, washed and resuspended in FACs buffer before acquisition. In the case of blood, erythrocyte lysis was performed after staining with ACK buffer. Samples were acquired in the Cytoflex S (Beckman Coulter) cytometer and sample data analysis was done using FlowJo X software (BD).

### Activation and proliferation with anti-CD3/CD28 Dynabeads

Naïve T cells (CD4^+^ CD25^-^ CD44^lo^ CD62L^hi^) from spleen and peripheral lymph nodes were sorted on the MoFlow FACS sorter and stained with Cell Trace Violet (CTV) following manufacturer’s instructions. 5x10^4^ CTV^+^ cells per well were cultured in a 96-well plate with Dynabeads^®^ Mouse T-Activator CD3/CD28 (Invitrogen) at a 2:1 beads/cell ratio and complete RPMI medium for 4 days. Cells were recovered at 12, 24, 48, 72, and 96 hours and stained for the detection of CD4, CD25, CD44, and CD69 and viability. Zombie NIR dye (Biolegend) and antibodies were diluted in PBS and cells were incubated with the mix for 15 min at room temperature, washed, fixed, and acquired in the Attune NxT Cytometer (Thermo Fisher). Data analysis was done using FlowJo X software.

For the proliferation index, division index, and precursor frequency calculations, the number of cells of G0, G1, G2, G3 …. were obtained and the following algorithms were used:

Division index = # of divisions/# of cells at start of culture

Proliferation index = # of divisions/# of cells that went into division

Precursor frequency = (division index/proliferation index) * 100

where:

# of divisions = (G1/2)*1 + (G2/4)*2 + (G3/8)*3 ….

# of cells at the start of culture = G0 + (G1/2) + (G2/4) + (G3/8) ….

# of cells that went into division = (G1/2) + (G2/4) + (G3/8) ….

### *In vitro* T cell differentiation with anti-CD3/CD28 Dynabeads

CD4^+^ CD25^-^ CD44^lo^ CD62L^hi^ Naϊve T cells from spleen and peripheral lymph nodes were sorted on the MoFlow FACs sorter and stained with CTV. 5 x10^4^ cells per well were cultured in a 96-well plate for 3 days with Dynabeads^®^ Mouse T-Activator CD3/CD28 (Invitrogen) at a 2:1 beads/cell ratio and complete RPMI medium supplemented with 10 ng/mL IL-12 (R&D Systems) and 10 µg/mL anti-IL4 (Biolegend) for Th1 differentiation and 1.5 ng/mL TGF-β1 (Peprotech), 20 ng/mL IL-6 (R&D Systems), 10 ng/mL IL-23 (R&D Systems), 10 µg/mL anti-IL-4 (Biolegend), and 10 µg/mL anti-IFN-γ (Biolegend) for Th17 differentiation. On day 3, cells were restimulated with 50 ng/mL PMA and 500 ng/mL ionomycin and treated with 5 µg/mL brefeldin A (GolgiPlug™, BD) for 5 hours at 37°C. Surface staining with viability dye and anti-CD4 and anti-CD25 was performed at room temperature for 15 min. Cells were fixed and permeabilized with 100 **μ**L of Foxp3/Transcription Factor Fix/Perm Solution (Tonbo) for 1 hour at room temperature. After permeabilization, Fc receptors were blocked with anti-mouse CD16/CD32 (Biolegend) for 20 min at room temperature. Intracellular staining with anti-IFNγ and anti-IL-17A was performed for 30 min at room temperature. Th1 and Th17 polarized cells were identified as CD4^+^ IFN-γ^+^ and CD4^+^IL-17A^+^, respectively, and samples were acquired in an Attune NxT cytometer (Thermo Fisher) and analyzed with FlowJo X software (BD, San Jose, CA).

### *In vitro* Th1 and Th17 polarization of MOG specific T cells

*Tgfbr3^fl/fl.^dLcKCre* mice (n=9) and *Tgfbr3^fl/fl.^* mice (n=8) were immunized with 150 μg of myelin oligodendrocyte glycoprotein 35-55 peptide (MOG_35-55_, GL Biochem Ltd.) emulsified in complete Freund’s adjuvant containing 500 μg of *Mycobacterium tuberculosis* (BD). Ten days following immunization, cells from spleen and peripheral lymph nodes of *Tgfbr3^fl/fl.^dLcKCre and Tgfbr3^fl/fl.^* mice were obtained. After RBC lysis, 5x10^6^ cells from *Tgfbr3^fl/fl.^dLcKCre* mice and *Tgfbr3^fl/fl.^* mice were cultured in a 48-well plate with 10 μg/mL MOG_35-55_ under either Th1 or Th17 polarizing conditions. Th1 polarizing conditions consisted of 10 ng/mL IL-12 (Biolegend) and 0.5 μg/mL anti-IL-4 (Biolegend) in IMDM complete culture media. Th17 polarizing conditions consisted of 20 ng/mL IL-6 (Tonbo Biosciences), 20 ng/mL IL-23 (Biolegend), 1 ng/mL TGF-β1 (Tonbo Biosciences), 10 μg/mL anti-IFN-γ (Biolegend), and 0.5 μg/mL anti-IL-4 (Biolegend) in IMDM complete culture media. After 3 days, 50 ng/mL of PMA (Biolegend), 500 ng/mL of ionomycin (Biolegend), and 5 μg/mL of brefeldin A (Biolegend) were added to each well and cells were incubated for 4 hours at 37°C (29). Cells were stained with Zombie NIR (Biolegend) followed by surface staining with anti-CD4 BV650 (Biolegend) and anti-CD5 PE (Biolegend). Permeabilization and intracellular staining with anti-IFN-γ APC (Biolegend) and anti-IL-17A AF488 (Biolegend) antibodies was then performed. Samples were acquired with an Attune Nxt flow cytometer (Thermo Fisher) and analyzed with FlowJo X software (BD).

### Induction of EAE

Active EAE model was induced as previously described (30). Briefly, mice were immunized with 150 μg of MOG_35-55_ (GL Biochem Ltd.) emulsified in complete Freund’s adjuvant containing 500 μg of *Mycobacterium tuberculosis* (BD). Mice also received an intraperitoneal injection of 200 ng pertussis toxin (List Biological Laboratories, INC.) immediately following immunization, as well as 2 days after immunization. Mice were scored daily to assess clinical symptoms of EAE for 30 days. The EAE scoring system ranges from a score of 0 to 6, as described below, with higher scores representing increased paralysis and disease severity (31).

The onset of disease presents with a partial loss of tail tone scored as a “1”. The disease progresses to a “2” when the tail is completely flaccid and does not respond to stimulation. A score of “3” is accompanied by the paralysis of one hind limb, but not both. A score of “4” is ascribed for complete paralysis of both hind limbs, but the ability to maneuver around the cage and access food and water using the fore limbs. Once a mouse does not have the ability to access food and water, or they have lost over 20% of their baseline body weight, the mouse is scored as a “5” and humanely euthanized. If a mouse is found dead in its enclosure, it is scored as a “6”. If a mouse dies during the observation period, or is euthanized, it is removed from analysis the day after death/euthanization.

### Passive EAE model

For passive induction of EAE, MOG_35-55_ reactive Th1 or Th17 cells were prepared as described previously (32, 33). Briefly, donor *Tgfbr3^fl/fl.^dLcKCre and Tgfbr3^fl/fl^* mice were immunized with 150 µg MOG_35-55_ in complete Freund’s adjuvant containing 500 μg of *Mycobacterium tuberculosis* (BD). Ten days following immunization, cells from peripheral lymph nodes and spleen were restimulated with 10 μg/mL MOG_35-55_ under either Th1 or Th17 polarizing conditions for 3 days. Th1 polarizing conditions consisted of 10 ng/mL IL-12 (Biolegend) and 0.5 μg/mL anti-IL-4 (Biolegend) in IMDM complete culture media. Th17 polarizing conditions consisted of 20 ng/mL IL-6 (Tonbo Biosciences), 20 ng/mL IL-23 (Biolegend), 1 ng/mL TGF-β1 (Tonbo Biosciences), 10 μg/mL anti-IFN-γ (Biolegend), and 0.5 μg/mL anti-IL-4 (Biolegend) in IMDM complete culture media. The differentiated cells were harvested, pooled, and dead cells were removed using a Ficoll gradient. These cells were used as “donor cells’’. The number of transferred donor cells was normalized based on the percentage of live CD4+ cells; sublethally irradiated (350 rads) naïve mice were injected with 5x10^6^ cells (Th1 or Th17) resuspended in 500 μL of PBS *via* intravenous tail injections. Recipient mice were also intraperitoneally administered 200 ng of the pertussis toxin same as for active EAE and they were scored daily to assess clinical symptoms of EAE for 30 days, as described above (34–36).

### Anti-TNP Ab response

Anti-TNP primary and secondary Ab response was determined by immunizing (primary) mice with 50 µg TNP-KLH (Sigma) in complete Freund’s adjuvant (CFA) followed by boost on day 21 with 50 µg TNP-KLH in incomplete Freund’s adjuvant (IFA). Mice were bled at baseline (before immunization), day 7, 14, and 21 after primary immunization, and day 28, 36, and 42 post boost. Levels of anti-TNP in serum were measured by ELISA using anti-TNP-BSA (1:4) as antigen. Responses for IgM, IgG1, IgG2c, and IgG3 anti-TNP were assayed.

### Statistical analysis

A nonparametric Mann-Whitney U test was performed from day 15 to day 30, for the *in vivo* experiments and independent sample t tests were conducted for *in vitro* assays. Prism (GraphPad) was used to perform the analysis.

## Results

### TβRIII deletion in peripheral T cells does not affect the homeostasis of CD4+ and CD8+ cells

**Figure 1:**
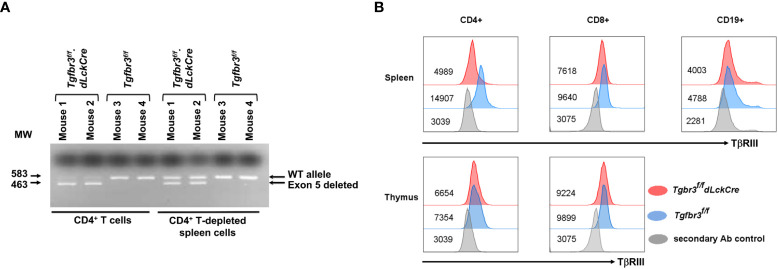
Inactivation of TβRIII in peripheral CD4 T cells in *Tgfbr3^fl/fl^.dLcKCre* mice. The TβRIII deletion was assessed by PCR and by flow cytometry on CD4^+^ T cells. **(A)** Representative electrophoresis gel for presence of *Tgfbr3* 5 (583 bp) or its deletion (463 bp) in CD4^+^ cells or CD4-depleted spleen cells from *Tgfbr3^fl/fl^.dLcKCre* and *Tgfbr3^fl/fl^* control mice. **(B)** Representative histograms for TβRIII expression on CD4^+^, CD8^+^, and CD19^+^ cells from spleen and thymus of *Tgfbr3^fl/fl^.dLcKCre* (red) and *Tgfbr3^fl/fl^* (blue) control mice, including the secondary antibody control (gray) for each population, median expression is represented next each histogram.

To investigate the function of the TβRIII protein in mature T lymphocytes, we bred the *Tgfbr3^fl/fl^* mouse with the distal *Lck* promoter *Cre* transgenic mice to generate *Tgfbr3^fl/fl^*.*dLckCre* mice (37, 38). PCR analysis of purified CD4^+^ T cells from spleen show a prominent 463 bp band, the product after deletion of exon 5, and a faint 583 bp band representing wild type (**Figure 1A**). In contrast, CD4^+^ T cell-depleted splenocytes, predominantly containing B cells and CD8^+^ T cells, presented the wild type (583 bp) and deleted (463 bp) bands, respectively. We next assessed the expression of TβRIII protein on the cell surface of CD4^+^ T cells, CD8^+^ T cells, and CD19^+^ B cells from peripheral tissue and blood by flow cytometry. On CD4^+^ T cells from all peripheral tissues (spleen, LN, MLN, and blood), we detected very little expression of TβRIII on CD4^+^ T cells from*Tgfbr3^fl/fl^*.*dLckCre* mice compared to *Tgfbr3^fl/fl^* controls (**Figure 1B** and data not shown). The low levels of TβRIII probably represent incomplete deletion that is evident in the PCR results. Although the TβRIII levels on CD8^+^ T cells from *Tgfbr3^fl/fl^*.*dLckCre* mice were lower than those from *Tgfbr3^fl/fl^* mice, its expression was clearly detected as compared to secondary Ab control staining. In the thymus, *Tgfbr3^fl/fl^* mice and *Tgfbr3^fl/fl^*.*dLckCre* had equivalent levels of TβRIII on CD4^+^ and CD8^+^ T cells, an expected result since the dLck promoter becomes active only in mature T cells in the periphery (37). A very small population of B cells express Lck and therefore TβRIII expression was mostly unaltered in *Tgfbr3^fl/fl^*.*dLckCre* mice. Furthermore, B cells express very low levels of TβRIII, as previously reported (17).

**Figure 2:**
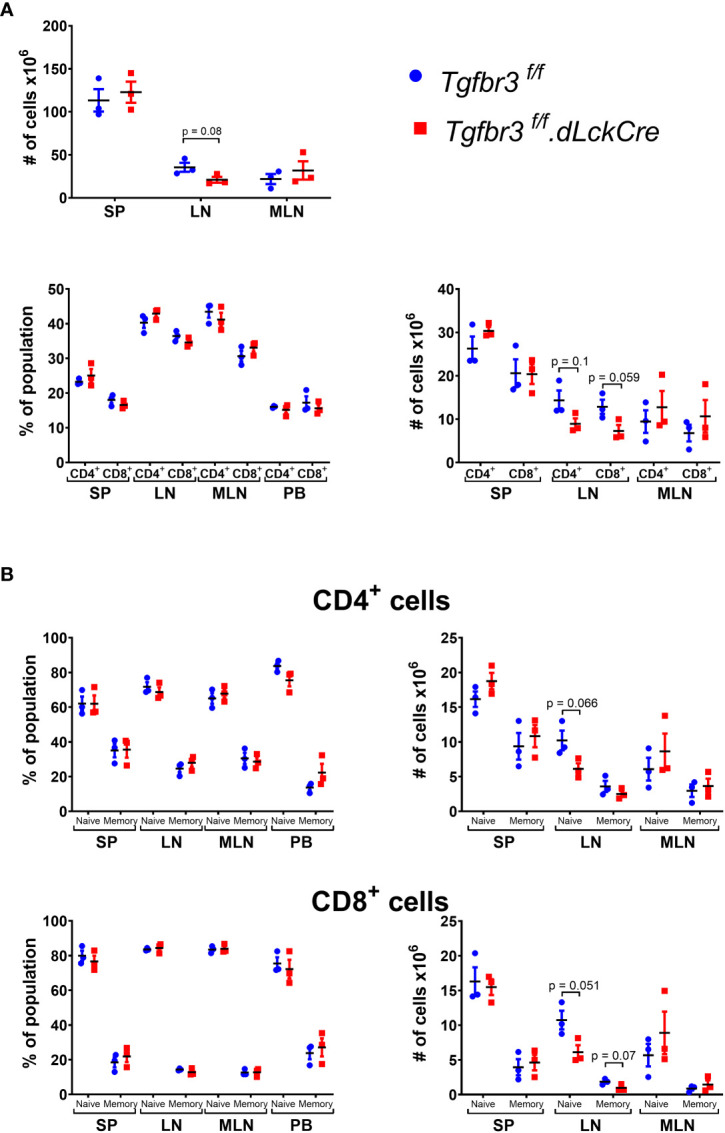
Evaluation of lymphocyte populations in *Tgfbr3^fl/fl^.dLckCre* mice at homeostasis. **(A)** Scatter plots show the of total number of cells in spleen (SP), peripheral lymph nodes (LN) and mesenteric lymph nodes (MLN) from *Tgfbr3^fl/fl^.dLcKCre* (red) and *Tgfbr3^fl/fl^* (blue) (upper graphs) and the percentages and absolute numbers of CD4^+^ and CD8^+^ populations in the secondary lymphoid organs and peripheral blood from (bottom graphs) of the same mouse strains. **(B)** Percentage of naïve (CD62Lhi/CD44lo) or memory population (CD62L-/CD44hi) from CD4^+^ and CD8^+^ T cells in spleen (SP), peripheral lymph nodes (LN) mesenteric lymph nodes (MLN) and peripheral blood from *Tgfbr3^fl/fl^ dLcKCre* (red) and *Tgfbr3^fl/fl^* (blue) control mice. Bar graphs show mean ± SEM. *p ≤ 0.05, **p ≤ 0.01, ***p ≤ 0.001. n = 3-4 mice, from 3 independent experiments.

TβRIII is differentially expressed on CD4 and CD8 T cell populations with increased expression on naïve and central memory CD4 T cell subpopulations (17), suggesting that TβRIII may be involved in T cell homeostasis. We did not observe any differences in frequencies or total numbers of CD4^+^ and CD8^+^ T cells, nor subpopulations of CD4^+^ T cells in peripheral lymphoid organs or blood (**Figure 2**, **Supplementary Figure 1**). The total number of cells in spleen (SP) and mesenteric lymph nodes (MLN) was similar in *Tgfbr3^fl/fl^*.dLckCre and *Tgfbr3^fl/fl^* mice, however, in peripheral lymph nodes (LN) there was a slight decrease in total cell numbers, as well as CD4^+^ T cells and CD8^+^ T cell numbers in *Tgfbr3^fl/fl^*.*dLckCre* compared to control *Tgfbr3^fl/fl^* mice (**Figure 2A**). This decrease in LN was primarily within the naïve CD4 and CD8 T cell subsets (**Figure 2B**).

### TβRIII null CD4 T cell mice develop more severe EAE

**Figure 3:**
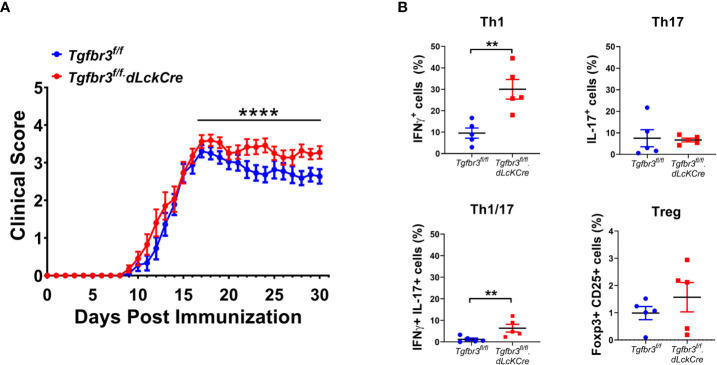
T cell specific TβRIII null mice develop more severe EAE after immunization with MOG_35-55_. **(A)** EAE clinical score of *Tgfbr3^fl/fl^.dLcKCre* (red, N=20) and *Tgfbr3^fl/fl^* (blue, n=18) mice immunized with MOG_35-55_ peptide in CFA. **(B)** Th17, Th1, Th17/1 and Treg cells in spinal cord of *Tgfbr3^fl/fl^ dLcKCre* (red) and *Tgfbr3^fl/fl^* (blue) mice at peak of disease. Littermate mice were immunized with 150 µg MOG_35-55_ peptide in CFA and clinical course of disease was determined. Mice with an EAE clinical score above 4 or a drop in weight of over 20%, mice were humanely euthanized. After a mouse was euthanized, it ceased to be scored and was therefore removed from future analysis. In **(B)** each dot represents an individual mouse. Data is mean ± SEM; Non-parametric t-test for **(A)** and parametric t-test for **(B)**. **=p<0.01,, ****=p<0.0001.

Autoreactive CD4^+^ T cells initiate the disease in EAE, the mouse model to study MS (34). Molecules that modulate strength of the CD4 T cell response and/or ability to differentiate into pathogenic effector populations such as Th1, Th17 directly impact both the development and severity of EAE disease (24). Thus, this *in vivo* model is a robust approach to determine to what extent TβRIII impacts CD4 T cell activation and/or differentiation to effector Th cells. We immunized female and male *Tgfbr3^fl/fl^*.dLckCre and *Tgfbr3^fl/fl^* mice with MOG_35-55_ and followed the development and progression of EAE over 30 days. We observed that *Tgfbr3^fl/fl^.dLckCre* mice developed EAE with significantly greater severity than *Tgfbr3^fl/fl^* mice, with a median clinical score of 3.28 and 2.76, respectively (**Figure 3A**). *Tgfbr3^fl/fl^.dLckCre* showed greater mortality than Tgfbr3*^fl/fl^* mice from EAE, however, overall survival as assessed by Kaplan-Meier analysis was not significantly different between the two groups (data not shown). It is important to note that the survival analysis only included mice that died spontaneously and none that were euthanized due to severe disease (more than 20% of loss in body weight or clinical score ≥ 3.5). Disease onset was not different between TβRIII null and controls (**Figure 3A**).

Th1 and Th17 cells contribute to EAE severity and Treg cells suppress disease (39), therefore we interrogated if increased disease severity in *Tgfbr3^fl/fl^*.dLckCre mice is associated with changes in these effector CD4 T cell populations. Mice were euthanized around the peak of disease, and the T cell populations in the spinal cord were analyzed for frequency of CD4^+^ T cells expressing IFN-γ, IL-17 or CD25 and FoxP3. *Tgfbr3^fl/fl^.dLckCre* mice had a significantly higher proportion of IFN-γ^+^ (Th1) and IFN-γ/IL-17 double positive cells in the spinal cord compared to *Tgfbr3^fl/fl^* mice (**Figure 3B**). No significant differences were observed between *Tgfbr3^fl/fl^.dLckCre* and *Tgfbr3^fl/fl^* mice for the proportion of IL-17^+^ (Th17) (**Figure 3B**). Although, the total numbers of CD4^+^ T cells in the spinal cord were not significantly different between *Tgfbr3^fl/fl^.dLckCre* and *Tgfbr3^fl/fl^* mice, TβRIII null mice had significantly higher numbers of Th1 (IFN-γ^+^) T cells in the spinal cord compared to controls (**Supplementary Figure 3**). We also performed an analysis of CD25^+^Foxp3^+^ Tregs (**Figure 3B**) and CD8^+^ T cells (not shown) and found no significant differences in the spinal cord of *Tgfbr3^fl/fl^*.dLckCre compared to *Tgfbr3^fl/fl^* mice with active EAE.

These results indicate that loss of TβRIII leads to expansion of Th effector cells expressing IFN-γ *in vivo*. This inference is supported by an independent experiment showing greater anti-trinitrophenyl (TNP) secondary IgG2c and IgG3, but not IgG1 and IgG2b, antibody response following immunization with TNP-KLH (**Supplementary Figure 2**). Class switch to IgG2c and IgG3 is promoted by IFN-γ expressing CD4 T cells (40).

### TβRIII loss does not affect activation and differentiation of CD4+ T cells *ex vivo*

To investigate the mechanisms underlying the increase in IFN-γ expressing Th cells contributing to the enhanced EAE and perhaps the IFN-γ dependent IgG class switch antibody response observed in TβRIII null mice, we performed a set of functional experiments.

**Figure 4:**
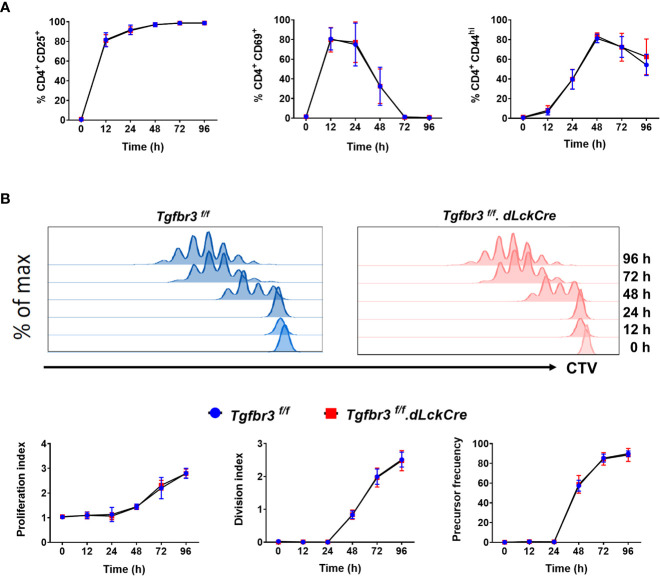
*In vitro* activation of CD4^+^T cells from *Tgfbr3^fl/fl^ and Tgfbr3^fl/fl^.dLckCre* mice. **(A**) Expression of the activation markers CD25, CD44 and CD69 was analyzed at 0, 12, 24, 48, 72, and 96 h on sorted naïve T cells stimulated with anti-CD3/anti-CD28. **(B**) Proliferation of naïve T cells from *Tgfbr3^fl/fl^* (blue) and *Tgfbr3^fl/fl^.dLcKCre* (red) mice was evaluated by CTV dilution. Histograms from a representative experiment are shown. Data are expressed as proliferation index, division index and precursor frequency for each time point (see methods). Graphs represent mean ± SEM (n = 5 independent experiments, 5 mice per group).

We previously reported that expression of TβRIII on CD4^+^ T cells is induced upon CD3 and CD28 crosslinking, concomitantly with increased expression of CD25, CD69, and CD44 (17), suggesting that this coreceptor could be functionally relevant for T cell activation and differentiation. Therefore, we stimulated CD4^+^ T cells with anti-CD3/CD28 and assayed for the induced expression of activation markers including CD25, CD44, and CD69 as a readout for T cell activation. We found no difference in induction and levels of expression of the three activation markers on CD4^+^ T cells from T*gfbr3^fl/fl^.dLckCre* and *Tgfbr3^fl/fl^* mice (**Figure 4A**). In addition, CD3/CD28 co-stimulation induced T cell proliferation measured by CTV dilution was equivalent between TβRIII null and wild type CD4^+^ T cells at all time-points between 24 to 96 hours of stimulation (**Figure 4B**). This was the case for proliferation index, division index and precursor frequency.

**Figure 5:**
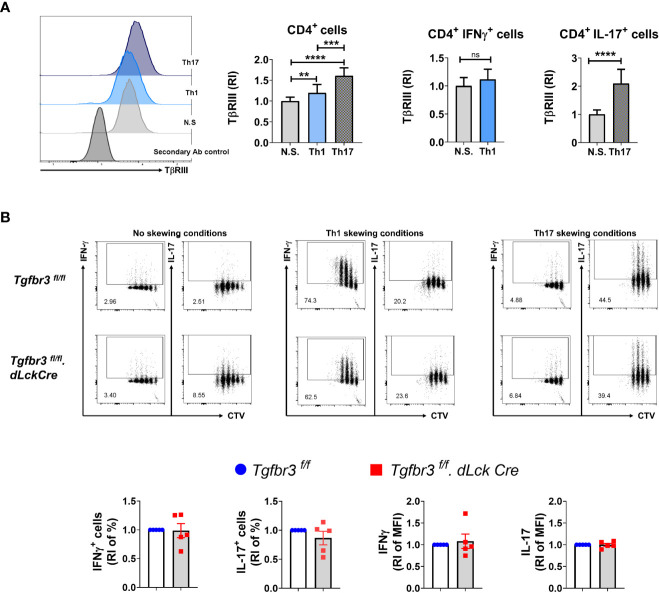
TβRIII on Th1 and Th17 cells *in vitro* differentiated CD4^+^ T cells. The expression of TβRIII was evaluated on CD4^+^ T cells under Th1, Th17 and non skewing (N.S) conditions. **(A)** TβRIII expression on CD4^+^ T cells under non skewing, Th1 and Th17 conditions. Representative histograms TβRIII expression on Th1, Th17, NS and secondary Ab control (left). Graphs of the increased TβRIII expression relative to non skewing conditions on CD4^+^, CD4^+^IFNγ^+^ and CD4^+^IL17^+^ T cells (right). **(B**) Representative dot plots of IFN-γ^+^ CTV^+^ and IL-17^+^ CTV^+^ live CD4^+^T cells following *in vitro* Th1 and Th17 differentiation from *Tgfbr3^fl/fl^.dLcKCre* (red) and *Tgfbr3^fl/fl^* (blue) mice; below are the graphs of IFN-γ or IL-17 increased percentage and median fluorescence relative to non skewing compared to Th1 and Th17 skewing conditions. Bar graphs show mean ± SEM. *p ≤ 0.05, **p ≤ 0.01, ***p ≤ 0.001. n =5 independent experiments for all conditions ****p<0.0001.

We have previously reported that expression of TβRIII is modulated by T cell activation. Interestingly, TβRIII expression inversely correlates with FoxP3 expression levels during *in vitro* iTreg differentiation in the presence of TGFβ (17). To determine if TβRIII expression is differentially modulated during polarization of CD4^+^ T cells into Th1 and Th17 cells, we isolated naïve CD4^+^ T cells and cultured them under appropriate skewing conditions. We observed that TβRIII expression was higher on CD4^+^ T cells cultured in either Th1 or Th17 polarizing conditions than non-skewed CD4^+^ T cells (**Figure 5A**). Furthermore, expression of TβRIII was greater on Th17 skewed cells compared to Th1 skewed cells. Focusing on only cytokine-expressing cells, we observed that IL-17^+^ and not IFN-γ^+^ expression was associated with enhanced upregulation of TβRIII expression. This led us to hypothesize that TβRIII might contribute to Th17 differentiation more than Th1 differentiation. However, in *ex vivo* Th differentiation cultures, naïve CD4^+^ T cells from *Tgfbr3^fl/fl^* and *Tgfbr3^fl/fl^*.*dLckCre* mice differentiated similarly under Th1 and Th17 skewing conditions (**Figure 5B**). There was a trend towards increase in IFN-γ^+^ cells in TβRIII null T cells, but the difference was not significant. We tested if TβRIII impact on Th differentiation is dependent cell division using CTV dilution assay. Here again we were unable to identify any difference between *Tgfbr3^fl/fl^*.dLckCre and *Tgfbr3^fl/fl^* CD4^+^ T cells (**Figure 5B**). Overall, these results indicate that under conventional *ex vivo* culture conditions, the activation and differentiation of naïve CD4^+^ T cells is not altered by the presence or absence of TβRIII.

### TβRIII null encephalitogenic Th17 cells induce more severe EAE

**Figure 6:**
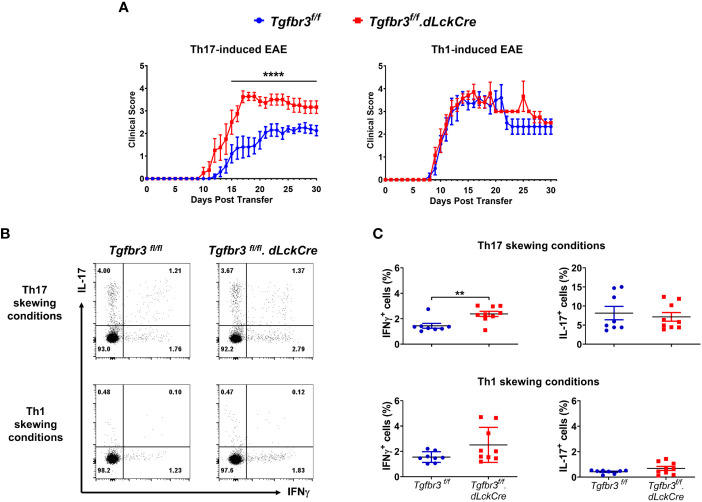
TβRIII regulates the ability of encephalitogenic Th17 to induce EAE. Encephalitogenic Th17 or Th1 cells were generated from *Tgfbr3^fl/fl^* (blue) or *Tgfbr3^fl/fl^.dLcKCre* (red) donor mice and transferred to naïve *Tgfbr3^fl/fl^* recipients. **(A)** Clinical scores of passive EAE after transfer of encephalitogenic Th17 and Th1 T cells from both strains into naïve wild-type recipients. The data is expressed as mean ± SEM. For Th17 transfer experiments: TβRIII control (n=10) and TβRIII null (n=8). For Th1 transfer experiments: TβRIII control (n=7) and TβRIII null (n=7). Non parametric ****p ≤ 0.0001. **(B, C)** Frequency of IL-17 and IFN-γ expressing Th cells in Th17 skewed or Th1 skewed cultures of T cells from MOG_35-55_ peptide immunized *Tgfbr3^fl/fl^* or *Tgfbr3^fl/fl^.dLcKCre* mice. **(B)** Representative dot plots (left) and scatter plots (right). Each dot in the scatter plot represents an individual mouse and data is mean ± SEM. Parametric student t-test **p ≤ 0.01.

As previously described, independent and overlapping cytokine-mediated mechanisms regulate pathogenicity of encephalitogenic Th1 and Th17 cells in their ability to passively induce EAE in naïve mice (32, 41, 42). Relevant to this, increased severity of EAE in TβRIII null mice was associated with a greater proportion and numbers of IFN-γ expressing Th cells in spinal cords (**Figure 3**). This led us to predict that TβRIII likely restrains pathogenicity of effector Th cells, particularly Th1 cells. To test for this, we generated encephalitogenic Th17 or Th1 cells by immunizing*Tgfbr3^fl/fl^*.*dLckCre* mice or *Tgfbr3^fl/fl^* mice, isolated *in vivo* primed/activated T cells and subsequent *ex vivo* antigen (MOG_35-55_) induced expansion in the presence of Th17 or Th1 skewing conditions. These encephalitogenic Th1 or Th17 cells were transferred into naïve mice and progression of EAE was determined. Recipients of TβRIII null Th17 cells developed EAE with earlier onset, beginning day 10, vs controls beginning day 12 (**Figure 6A**). TβRIII null Th17 cells also induced EAE with much greater severity than controls. There was no mortality in either of the recipient groups. In contrast to Th17 transfers, there was no difference between wild type and TβRIII encephalitogenic Th1 cells to induce EAE and the majority of recipients died within the 30-day time period (**Figure 6B**). We characterized the encephalitogenic Th17 and Th1 cells that were *in vivo* primed, and *ex vivo* restimulated/skewed. We determined that CD4^+^ T cells from TβRIII null mice skewed under Th17 conditions with MOG_35-55_ had an expanded proportion of IFN-γ expressing cells but equivalent IL-17^+^ cells to controls (**Figure 6C** upper graph). There was no difference between TβRIII null and controls for cells skewed under Th1 conditions (**Figure 6C** bottom graph). Overall, these data indicate that TβRIII restrains the pathogenicity of encephalitogenic Th17 cells likely by a mechanism regulating the transition to IFN-γ expressing Th cells.

## Discussion

TβRIII is expressed on CD4^+^ and CD8^+^ T cells at all stages of development, however its role in these populations has been elusive. Our group previously demonstrated from experiments in fetal thymic organ cultures that TβRIII plays a protective role in thymocyte survival. Furthermore, we showed that the expression of the coreceptor is upregulated upon T cell activation and therefore we hypothesized that TβRIII modulates processes downstream of TCR signaling. However, the ability to examine the function of TβRIII has been a challenge, as this coreceptor plays a key role in several developmental processes during fetal life such that its developmental knockout results in neonatal mortality (5). To overcome this limitation, we generated a *Tgfbr3^fl/fl^* mouse and bred it with the *dLck.Cre* mouse for conditional deletion of TβRIII in mature T cells (37). We confirmed that Cre-mediated deletion was very effective by PCR analysis of genomic DNA for Cre-mediated deletion of *Tgfbr3* exon 5. In CD4^+^ T depleted spleen cells, two bands were observed: the lower band likely corresponds to CD8^+^ T cells (delete exon 5), and the upper band attributable to B cells (wild type band). CD4^+^ T cells were not further segregated into subpopulations since they all express high levels of TβRIII (17).

Flow cytometry analysis confirmed that TβRIII expression was selectively abrogated in CD4^+^ T cells (**Figure 1B**) and moderately on CD8^+^T cells, but not in thymic CD4^+^ T cells and CD19^+^ B cells, as the *Lck* distal promoter is selectively active in late mature T cells. *Tgfbr3* exon 5 deletion did not result in complete protein ablation, and although we cannot exclude that the remaining expression of TβRIII is functional, its dramatic decrease in expression observed in *Tgfbr3^f/f^.dLckCre* mice was sufficient to increase the severity of EAE induced by active immunization with MOG_35-55_ peptide or by passive immunization with encephalitogenic Th17 cells.

Although we did not find significant differences in the proportion of T cell subsets between *Tgfbr3^fl/fl^.dLckCre* and *Tgfbr3^fl/fl^* mice, there was a trend towards a decrease in LN CD4^+^ and CD8^+^ T cell numbers, which was not present in spleen and MLN, suggesting a possible LN-specific homing defect. Consistent with our previous report, TβRIII was differentially expressed between subpopulations of T cells, with greater levels on CD4^+^ compared to CD8^+^ T cells (17). This is especially relevant given that this receptor modulates cell migration and metastasis in different cancer type cells (3). We have previously shown that TβRIII is upregulated upon TCR crosslinking in concomitantly to other activation markers, such as CD69, CD25 and CD44 (17). Therefore, we speculated that this coreceptor might be involved in T cell activation. However, we found that under non-skewing conditions, T cell activation was not significantly affected by the absence of TβRIII.

Given the role of TβRIII in the modulation of TGFβ signaling (43) and our recent report showing that the expression of TβRIII is decreases with increase in Foxp3 expression during induced-Treg (iTreg) culture conditions (17), we predicted that this correceptor may modulate differentiation of naive CD4 T cells to Th17 and/or Th1 effector cells. The differentiation of naive CD4^+^ T cells following under appropriate polarizing conditions induced greatly elevated expression of TβRIII on Th17 cells, at levels greater than that on Th1 cells. However, the loss of TβRIII had no effect on the ability of naive T cells to differentiate into Th1 and Th17 under *in vitro* polarizing conditions. It is important to appreciate that TβRIII interacts with several different molecules; these include activins, inhibins, bone morphogenic proteins, in addition to TGFβs (1). The *in vitro* culture conditions do not replicate any of these interactions and therefore does exclude the possibility that TβRIII plays a role in Th1 and Th17 functional activities *in vivo.* In autoimmune diseases, including MS and its mouse model EAE, the importance of Th1 and Th17 cells in the pathogenesis of disease is well established (32). We found that TβRIII null mice developed more severe EAE than littermate controls. The severity of EAE was associated with an expanded proportion of Th1 and Th17/1 (IFN-γ^+^IL-17^+^) cells but not Th17 cells. The Th17/1 cells represent Th17 cells undergoing transition from Th17 to Th1, a process defined as plasticity (44). We and others have shown that these Th17/1 cells likely contribute more to pathogenicity in MS and EAE (45–47). The expanded Th1 cells in the spinal cord of TβRIII null mice may represent a function of TβRIII to restrict the differentiation of naïve CD4^+^ T cells into Th1 cells. This concept is supported by the observation that class switch of antibody to IFNγ-dependent isotypes (IgG2c and IgG3) was significantly greater in TβRIII null mice, although it was moderately observed for IgG1 and IgG2b.

Passive transfer of *in vivo* generated, *ex vivo* expanded encephalitogenic Th17 cells from TβRIII null mice induced significantly greater EAE than controls. The enhanced disease was not due to differences in numbers of transferred Th17 cells, but there was a small but significantly greater number of IFN-γ expressing cells generated from TβRIII null donors under Th17 conditions. It is unlikely that the increased pathogenicity is merely due to the presence of IFN-γ expressing Th cells as we did not observe any difference for Th1 transfers between TβRIII null and controls. This indicates to us that in the absence of TβRIII, T cells receiving Th17 environmental cues are more pathogenic. It is well appreciated that specific cues can shift the pathogenicity balance of Th17 cells (44, 48, 49).

The original view of the function of TβRIII as merely an accessory coreceptor for augmenting signals from TβRII-TβRII heterodimer needs to be reevaluated. TβRI or TβRII deficiency in CD4 cells results in the development of spontaneous autoimmunity in mice (18–20). If the main role of TβRIII is to function as a co-receptor for TβRI and TβRII, we would expect mice lacking TβRIII on CD4^+^ T cells to also exhibit spontaneous autoimmunity and death. However, we have studied the*Tgfbr3^fl/fl^.CD4Cre* mouse, which lacks TβRIII in all CD4^+^ T cells, and this mouse does not develop spontaneous autoimmunity up to 18 months of age, data not shown. Additionally, when we crossed this mouse with a *dLckCre* mouse to generate a *Tgfbr3^fl/fl^.dLckCre* mouse, which deletes TβRIII on mature T cells, no hyperactivation was observed as reported for TβRII null mice generated in a similar (38).

Results showed that TβRIII in T cells functions to restrict the development of pathogenic effector cells necessary for disease progression and severity in MOG_35-55_ immunization induced active EAE disease. This is evident by the fact that *Tgfbr3^fl/fl^.dLckCre* mice developed significantly more severe EAE than *Tgfbr3^fl/fl^* mice by both immunization with MOG_35-55_ and induction *via* the passive transfer of Th17 polarized T cells. The increase in disease severity in *Tgfbr3^fl/fl^.dLckCre* mice was also associated with an increased number and proportion of IFN-γ producing CD4^+^ T cells present in the spinal cord. However, there was no difference in IL-17 positive cells in the spinal cord of mice with EAE.

The passive transfer of Th1 polarized cells, regardless of the presence of TβRIII on T cells, resulted in rapid onset of severe disease and high mortality. This high level of mortality and disease was present despite the relatively low level of pathogenic T cell populations seen in culture prior to transfer compared to Th17 transfers. Additionally, the same level of sub-lethal radiation was applied to both Th1 and Th17 recipients and there was no mortality observed in Th17 transfer recipients. This may suggest a high level of pathogenic expansion in the Th1 passive transfer recipient mice regardless of the presence of TβRIII (50). The *in vitro* MOG restimulation/polarization data also suggests that TβRIII has no effect on the ability of naïve T cells to polarize towards Th1. Although the possibility cannot be excluded entirely, EAE induced by passive transfer of encephalitogenic Th1 cells from TβRIII sufficient and TβRIII null mice were indistinguishable even when the numbers of T cells transferred were reduced to as low as 2x10^6^ cells (data not shown). This indicates to us that the role of TβRIII in Th1 generation and/or pathogenicity may be subtle or needs to be addressed with other disease models.

The passive transfer Th17 EAE experiments showed that naïve recipient mice developed more severe disease after being injected with MOG-specific CD4^+^ T cells lacking TβRIII which had undergone Th17 polarization. However, investigation of these cells prior to transfer suggests that the primary cytokine produced by Th17 cells, IL-17, is likely not responsible for the difference in disease. The percentage of IL-17 producing cells was no different in cells lacking TβRIII and wild type in Th17 polarized MOG-restimulated cultures. Instead, MOG-specific CD4^+^ T cells lacking TβRIII which had undergone Th17 polarization produced a significantly higher proportion of the cytokine IFN-γ than cells from donor wild type mice. This supports our data showing increased IFN-γ expressing cells in the spinal cord of mice with active EAE.

These results suggest that MOG restimulated Th17 polarized CD4^+^ T cells lacking TβRIII may have undergone what is known as plasticity. Th17 cells with appropriate cytokine signals, such as IL-23 and IL-12 can transdifferentiate into IFN-γ producing Th1 cells (51). During conversion, the IL-17 expressing CD4^+^ T cells go through an intermediate stage expressing both IFN-γ and IL-17; in fact, it was observed that TβRIII null EAE mice had a greater proportion of the double cytokine producing cells than TβRIII sufficient mice [31]. Th1 cells generated from Th17 cells are also defined as ex-Th17 cells (27, 52, 53). Other effector populations such as Th1 and Th2 cells are fixed in their lineage and are thought to be unable to undergo this process of change to other effector cell populations (27). When Th17 cells begin to downregulate the expression of the master regulator RORγt and begin to upregulate the master regulator T-bet, they begin to take on the profile of Th1 cells and produce IFN-γ. The transdifferentiation of Th17 to Th1 cells is important for normal immune system function, such as promoting anti-tumor growth (54). However, there is evidence that suggests Th17 cells that transdifferentiate into Th1 cells can be particularly pathogenic. For example, the so-called ex-Th17 cells that produce IFN-γ are thought to be the main drivers of intestinal pathology in humans with inflammatory bowel disease and colitis (55, 56). Additionally, evidence suggests that ex-Th17 cells produce higher levels of cytokines than classical Th1 or Th17 cells and are also resistant to Treg suppression of proliferation and cytokine production (53).

## Data availability statement

The raw data supporting the conclusions of this article will be made available by the authors, without undue reservation.

## Ethics statement

The animal study was reviewed and approved by the "Comité Interno para el Cuidado y Uso de Animales de Laboratorio, (CICUAL), Protocol #176 at the Biomedical Research Institute, UNAM, and the UAB Institutional Animal Care and Use Committee (IACUC).

## Author contributions

GS and CR contributed to conception and design of the study. SOF, ROA, and SJD wrote the first draft of the manuscript. SOF, SJD, CMS, NAD, SMO, MSL, RC, PDS, NY, and ASL performed the experiments. SOF, ROA, and SJD organized the database and analyzed data. GS and CR wrote the final version of the manuscript. All authors contributed to the article and approved the submitted version.

## Supplementary material

Supplementary Figure 1 Gating strategy for the evaluation of lymphocyte populations in *Tgfbr3^fl/fl^.dLckCre* mice. CD4^+^ T cell subsets analysis was performed using the singlet cells, lymphocyte live cells (live and dead blue negative) gating. Then, cells were further gated for CD4^+^ and CD8^+^ and Naive Vs Memory subsets were evaluated using CD62L Vs CD44 markers. Finally, CCR7 analysis was performed from naive and memory subsets. CCR7 positive gate was adjusted based on the FMO controls of each subpopulation.

Supplementary Figure 2 Class switch to IgG1, IgG2b, IgG2c and IgG3 antibody secondary response in *Tgfbr3^fl/fl^.dLckCre* mice. Graphs of anti-trinitrophenyl (TNP) secondary IgM, IgG1, IgG2b, IgG2c and IgG3, antibody response following immunization with TNP-KLH in *Tgfbr3^fl/fl^.dLcKCre* (red) or *Tgfbr3^fl/fl^* (blue) littermate control mice. Bar graphs show mean ± SEM. *p ≤ 0.05, **p ≤ 0.01, ***p ≤ 0.001 (n =4).

Supplementary Figure 3 Absolute numbers of Th cells infiltrating the spinal cord in EAE model. Th1, Th17, Th1/17 cells in the spinal cord of *Tgfbr3^fl/fl^ dLcKCre* (red) and *Tgfbr3^fl/fl^* (blue) mice at peak of disease. Data represent mean ± SEM; *p ≤ 0.05, **p ≤ 0.01. (n = 5 mice for each strain).
