# Supplementary material for: Transforming growth factor receptor III (Betaglycan) regulates the generation of pathogenic Th17 cells in EAE
Source: Front Immunol. 2023 Feb 6;14:1088039. doi: 10.3389/fimmu.2023.1088039 (PMC9968395; doi:10.3389/fimmu.2023.1088039)
Supplement: Supplementary file 1 [file Table_1.docx]

**Table 1:** Antibodies used for flow cytometry experiments

| **Reagent** | **Source (Brand)** | **Identifier (#Catalog)** |
| --- | --- | --- |
| anti mouse TβRIII | R&D Systems | AF5034 |
| donkey anti goat IgG (H+L) Cross-Adsorbed Secondary Antibody, AF488 | Invitrogen | A11055 |
| anti mouse CD4 APC | Biolegend | 100412 |
| anti mouse CD25 PECy5 | Biolegend | 102010 |
| anti mouse/human CD44 PE | Biolegend | 103008 |
| anti mouse CD62L APC Cy7 | Biolegend | 104428 |
| anti mouse CCR7 AF700 | Invitrogen | 56-1971-82 |
| anti mouse CD8 BV 605 | BD Horizon | 563152 |
| anti mouse CD19 PercPcy5.5 | Tonbo Biosciences | 65-0193-U100 |
| anti mouse IFNγ BV510 | Biolegend | 505841 |
| anti mouse IL-17A PE | Biolegend | 506904 |
| anti mouse CD4 BV650 | Biolegend | 100546 |
| anti mouse CD5 PE | Biolegend | 100608 |
| anti mouse IFNγ APC | Biolegend | 505810 |
| anti mouse IL-17A Alexa Fluor 488 | Biolegend | 506910 |
| Live/Dead Blue | Invitrogen | L23105 |
| Zombie NIR | Biolegend | 423106 |
