# Supplementary figures and images for: Transforming growth factor receptor III (Betaglycan) regulates the generation of pathogenic Th17 cells in EAE
Source: Front Immunol. 2023 Feb 6;14:1088039. doi: 10.3389/fimmu.2023.1088039 (PMC9968395; doi:10.3389/fimmu.2023.1088039)

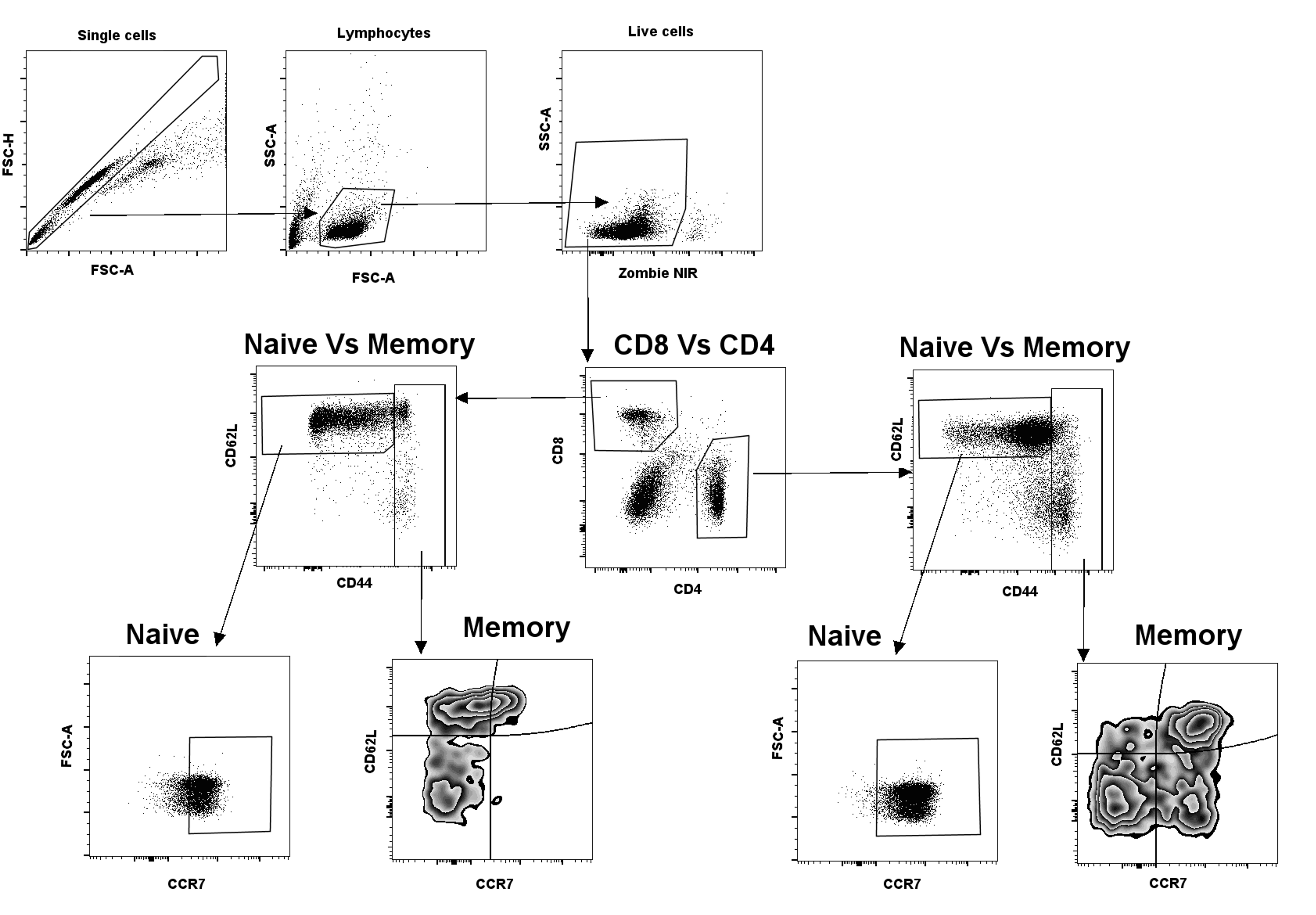

Supplement: Supplementary Figure 1 — Gating strategy for the evaluation of lymphocyte populations in Tgfbr3fl/fl.dLckCre mice. CD4+ T cell subsets analysis was performed using the singlet cells, lymphocyte live cells (live and dead blue negative) gating. Then, cells were further gated for CD4+ and CD8+ and Naive Vs Memory subsets were evaluated using CD62L Vs CD44 markers. Finally, CCR7 analysis was performed from naive and memory subsets. CCR7 positive gate was adjusted based on the FMO controls of each subpopulation. [file Image_1.tif]

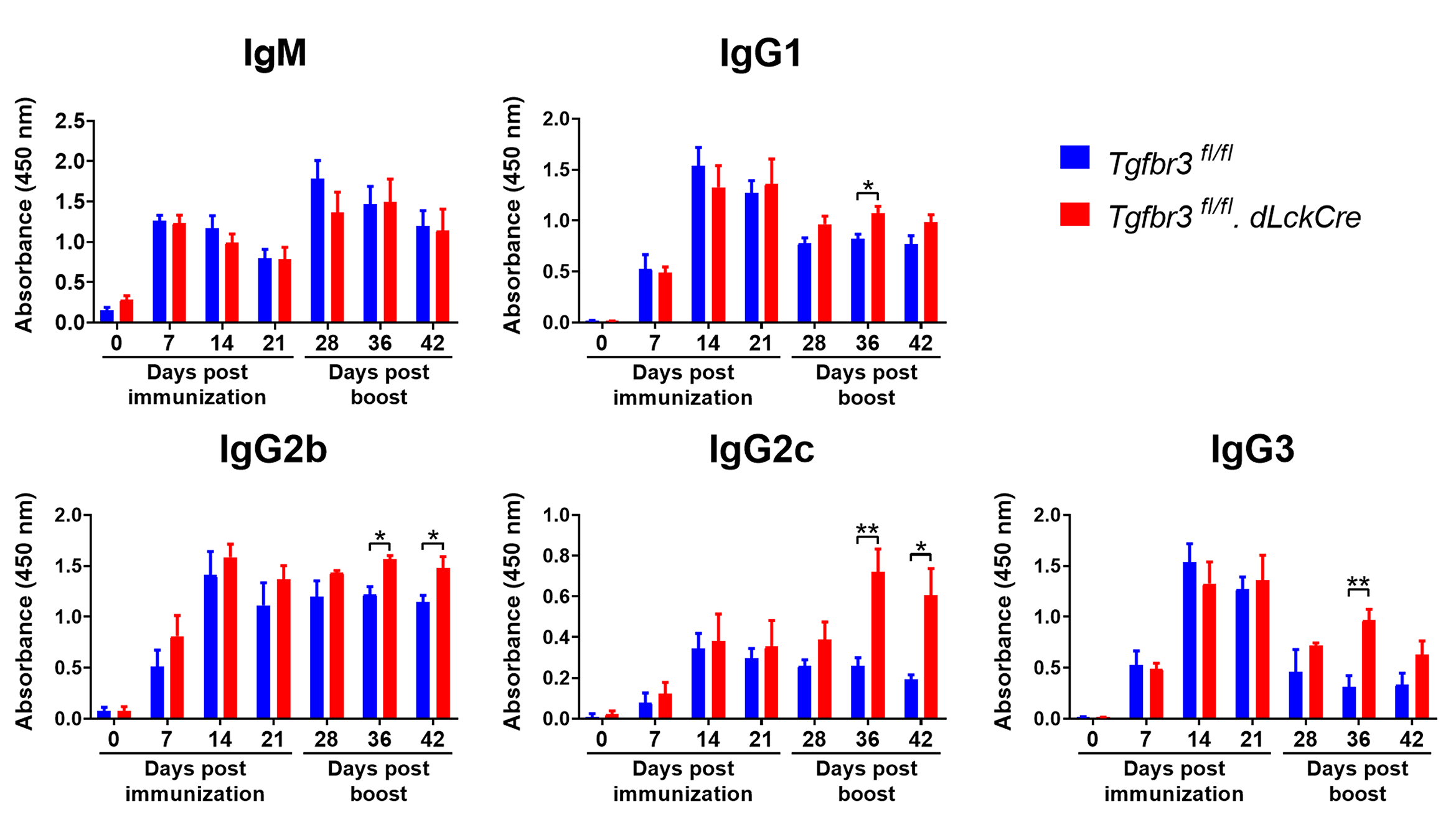

Supplement: Supplementary Figure 2 — Class switch to IgG1, IgG2b, IgG2c and IgG3 antibody secondary response in Tgfbr3fl/fl.dLckCre mice. Graphs of anti-trinitrophenyl (TNP) secondary IgM, IgG1, IgG2b, IgG2c and IgG3, antibody response following immunization with TNP-KLH in Tgfbr3fl/fl.dLcKCre (red) or Tgfbr3fl/fl (blue) littermate control mice. Bar graphs show mean ± SEM. *p ≤ 0.05, **p ≤ 0.01, ***p ≤ 0.001 (n =4). [file Image_2.tif]

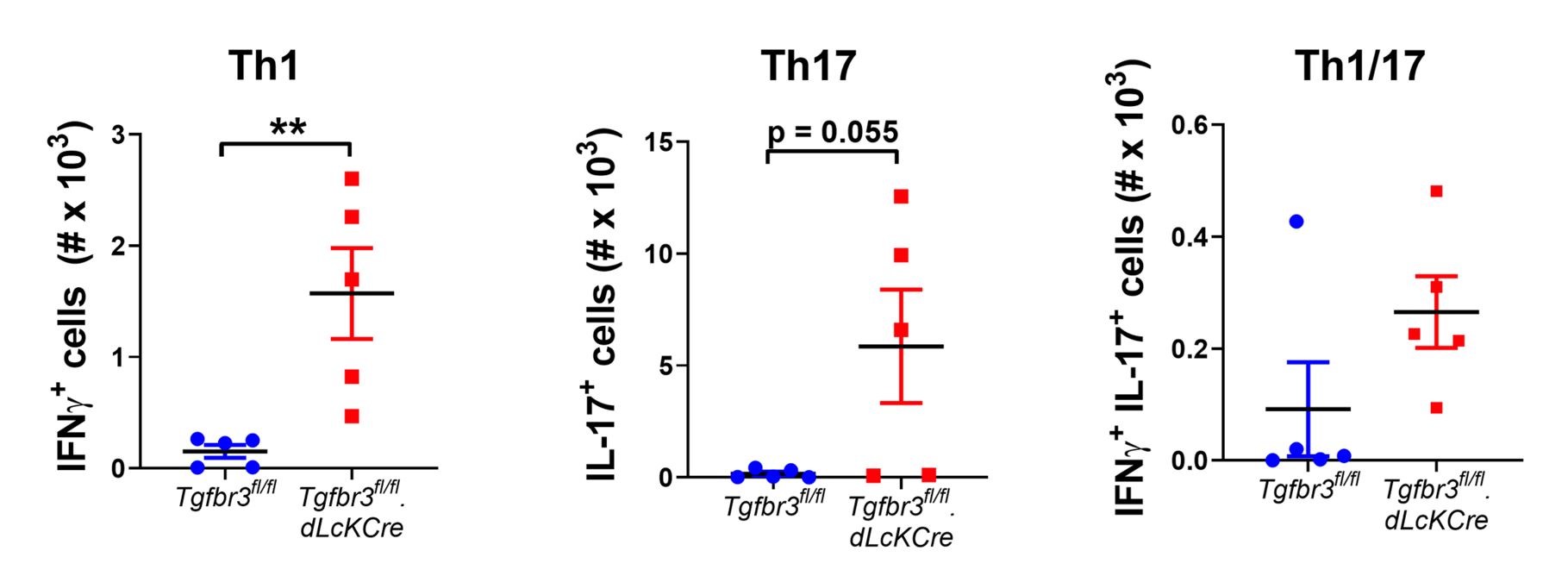

Supplement: Supplementary Figure 3 — Absolute numbers of Th cells infiltrating the spinal cord in EAE model. Th1, Th17, Th1/17 cells in the spinal cord of Tgfbr3fl/fl dLcKCre (red) and Tgfbr3fl/fl (blue) mice at peak of disease. Data represent mean ± SEM; *p ≤ 0.05, **p ≤ 0.01. (n = 5 mice for each strain). [file Image_3.tif]
